# Psychological distress as a central mediator of suicidal ideation among Brazilian veterinarians: A study of occupational stress, compassion fatigue, coping strategies, and workplace environment using structural equation modeling

**DOI:** 10.14202/vetworld.2026.604-618

**Published:** 2026-02-17

**Authors:** Bianca Stevanin Gresele, Jefferson Luiz Pereira, Alexandre Redson Soares da Silva, Karen Scavacini, Helena C. Lyrio-Carvalho, Sofia Marques Viana Ulisses, Anderson da Silva Rosa

**Affiliations:** 1 Graduate Program in Clinical Psychology, Department of Clinical Psychology, Pontifícia Universidade Católica de São Paulo (PUC), São Paulo 05015-901, SP, Brazil; 2 Graduate Program in Veterinary Sciences in the Semi-arid Region and College of Veterinary Medicine, Agricultural Sciences Campus, Universidade Federal do Vale do São Francisco (UNIVASF), Petrolina 56304-917, PE, Brazil; 3 Instituto Vita Alere, São Paulo, 04066-002, SP, Brazil; 4 Graduate School of Education, University of California - Riverside, Riverside 92521, CA, USA; 5 Collective Health Department, Escola Paulista de Enfermagem, Universidade Federal de São Paulo (UNIFESP), São Paulo 04024-002, SP, Brazil

**Keywords:** compassion fatigue, coping strategies, occupational stress, psychological distress, small animal veterinarians, structural equation modeling, suicidal ideation, workplace environment

## Abstract

**Background and Ai:**

Veterinarians experience disproportionately high levels of psychological distress and suicidal ideation compared with other professional groups. Occupational stress (OS), compassion fatigue, workplace environment (WE), and coping strategies have been identified as relevant risk or protective factors; however, their interrelationships remain insufficiently explored, particularly in Brazil. This study aimed to examine the direct and indirect pathways linking occupational psychosocial factors to suicidal ideation among Brazilian veterinarians working in companion-animal practice, with psychological distress conceptualized as a central mediating mechanism.

**Materials and Method:**

A nationwide cross-sectional survey was conducted between June and July 2022 involving 1,472 veterinarians exclusively engaged in small animal practice in Brazil. Participants completed validated self-report measures assessing psychological distress (Kessler-6), suicidal ideation, coping strategies, compassion fatigue, OS, and WE. Data were analyzed using partial least squares structural equation modeling (PLS-SEM) with 5,000 bootstrap resamples. Direct, indirect, and mediating effects were estimated, and model fit, reliability, and validity were assessed. Ethical approval was obtained from the Research Ethics Committee of Inspirar College (protocol no. 31645220.4.0000.5594).

**Result:**

Psychological distress emerged as the strongest predictor of suicidal ideation (β = 0.40; p < 0.001), explaining 20.6% of its variance. OS showed a substantial positive association with psychological distress (β = 0.47; p < 0.001), followed by compassion fatigue (β = 0.15; p < 0.001) and WE (β = 0.07; p = 0.028). Coping strategies exerted a significant protective effect on psychological distress (β = −0.26; p < 0.001) and suicidal ideation (β = −0.08; p = 0.013). Indirect effects confirmed that psychological distress mediated the associations between OS, compassion fatigue, WE, and suicidal ideation. Younger age and female gender were indirectly associated with higher suicidal ideation through increased psychological distress.

**Conclusio:**

Psychological distress plays a central mediating role in the relationship between occupational psychosocial factors and suicidal ideation among Brazilian veterinarians. Interventions targeting OS reduction, emotional support, and the promotion of adaptive coping strategies may substantially mitigate suicide risk in veterinary clinical practice.

## INTRODUCTION

In recent decades, veterinary medicine has been increasingly affected by psychosocial risks that substantially compromise professionals’ mental health. International evidence consistently demonstrates a high prevalence of psychological distress, burnout, and suicidal ideation among veterinarians, frequently exceeding levels reported in other health professions and in the general population [1–4]. Key risk factors include emotional overload associated with animal care, repeated exposure to euthanasia procedures, pressure from animal owners, occupational isolation, and ready access to potentially lethal means, particularly medications used in veterinary practice [5, 6]. Although this topic remains comparatively underexplored in Brazil, emerging research indicates that veterinarians are often exposed to markedly unfavorable working conditions. These include long working hours, multiple employment relationships, remuneration below the national average for professions requiring higher education, and limited social recognition [7, 8]. Such conditions are further aggravated by the fragility of occupational health policies within the veterinary sector [9].

Psychological distress, defined as a persistent state of emotional burden, is commonly associated with symptoms of anxiety, depression, mental fatigue, and hopelessness, and may progress to more severe outcomes such as emotional exhaustion and suicidal ideation [10, 11]. Suicidal ideation represents a critical warning sign, characterized by recurrent thoughts of death, disappearance, or self-harm, although these thoughts do not necessarily culminate in action. This condition reflects intense psychological suffering and warrants clinical attention. In contrast to suicidal behavior, which involves the enactment of a potentially lethal act, suicidal ideation denotes an earlier yet still pivotal stage within the suicidality continuum [12, 13]. Evidence suggests that veterinarians report suicidal thoughts more frequently than the general population, with Brazilian studies documenting elevated levels of psychological distress and recurrent death-related cognitions [14, 15]. Mental health outcomes in this population arise from the complex interaction between individual vulnerabilities and occupational as well as organizational stressors.

Coping strategies, understood as cognitive and behavioral efforts used to manage stressful situations, play a central role in this context and encompass a variety of practices that individuals may independently adopt in response to occupational stress (OS). Maladaptive strategies, including avoidance, denial, and substance use, have been linked to burnout and mental disorders, whereas adaptive strategies, such as seeking social support and engaging in positive reappraisal, have demonstrated protective effects [16–19].

Compassion fatigue, resulting from prolonged exposure to animal suffering and emotionally demanding interactions with owners, constitutes another important factor associated with emotional exhaustion, suicidal ideation, and intentions to leave professional practice [20–23], although evidence among Brazilian veterinarians remains limited [24]. Adverse workplace environments (WE) characterized by high emotional demands, restricted autonomy, inadequate recognition, and unsupportive institutional cultures have been consistently associated with psychological distress and suicidal ideation among healthcare professionals, including veterinarians [25–27]. Individual characteristics further modulate vulnerability, with women reporting higher levels of emotional symptoms and compassion fatigue, and younger or early-career professionals exhibiting greater psychological distress and less developed adaptive coping repertoires [28, 29]. Despite the recognized importance of these psychosocial factors, most existing studies have examined them in isolation or through descriptive designs, thereby limiting a comprehensive understanding of how occupational stressors, emotional processes, and coping resources jointly influence suicidal ideation [30, 31].

Despite increasing attention to veterinarians’ mental health, several critical conceptual and empirical gaps persist. Most existing studies have relied on descriptive or bivariate approaches, limiting insight into how occupational demands, emotional processes, and personal resources interact simultaneously within an integrated framework. Although the Job Demands–Resources (JD-R) model has been widely applied to explain how job demands and resources influence psychological outcomes, its application in veterinary medicine has rarely incorporated suicide-related outcomes as endogenous variables or tested complex mediational pathways [32, 33]. Similarly, the Interpersonal Theory of Suicide (ITS) emphasizes the role of proximal psychological states that intensify suicidal ideation over time; however, empirical applications of ITS in occupational contexts, particularly among veterinarians, remain limited [34, 35]. Importantly, few studies have explicitly examined psychological distress as a central mediating mechanism linking occupational stressors, compassion fatigue, WE, and coping strategies to suicidal ideation within a single structural model. This lack of integrative, theory-driven analyses constrains a comprehensive understanding of how distal occupational exposures translate into suicidal ideation through proximal emotional processes in veterinary professionals.

In response to these gaps, the present study aimed to investigate the direct and indirect relationships between OS, compassion fatigue, WE, coping strategies, psychological distress, and suicidal ideation among veterinarians working in small-animal practice in Brazil. Grounded in the JD-R model and ITS, this study specifically aimed to test psychological distress as a central mediating pathway through which occupational and organizational factors influence suicidal ideation. By applying partial least squares structural equation modeling (PLS-SEM), this study sought to develop an integrated explanatory model capable of clarifying the complex psychosocial mechanisms underlying suicidal ideation in veterinary medicine and to inform targeted, evidence-based prevention strategies.

## MATERIALS AND METHODS

### Ethical approval

The study was approved by the Ethics Committee for Research with Human Beings of Inspirar College (substantiated opinion No. 31645220.4.0000.5594), registered with the National Research Ethics Commission (CONEP) and linked to the Brazilian Ministry of Health. All procedures complied with Resolution CNS 466/2012, which regulates research involving human participants in Brazil, and adhered to the principles of the Declaration of Helsinki.

Participation was entirely voluntary and anonymous. Before accessing the online questionnaire, all potential participants received an electronic information sheet detailing the study objectives, potential risks and benefits, the voluntary nature of participation, confidentiality safeguards, and data-protection measures. Only individuals who confirmed their understanding and provided electronic informed consent were permitted to proceed. Participants were informed of their right to withdraw from the study at any time without justification or consequences.

Because the questionnaire included items addressing psychological distress and suicidal ideation, additional ethical safeguards were implemented. Participants were informed in advance that some questions could be sensitive or emotionally distressing and were allowed to skip any item they did not wish to answer. Upon completion of the survey, respondents were automatically provided with mental health support resources, including direct links to the Centro de Valorização da Vida (CVV), local mental health services, and emergency contacts, in accordance with World Health Organization recommendations for research involving suicide-related content. No personal identifiers, including names, email addresses, IP addresses, or clinic affiliations, were collected.

All data were stored on secure, encrypted servers with access restricted to authorized members of the research team. Approval was granted for the exclusive use of anonymized and aggregated data for scientific purposes. No financial incentives were offered, and no conflicts of interest related to participation were reported. All ethical standards for research involving human subjects were strictly observed, ensuring participant safety, confidentiality, and respect for autonomy throughout the study.

### Study period, location, and design

This nationwide cross-sectional study was conducted between June 29 and July 25, 2022, and included male and female veterinarians working exclusively with companion animals across Brazil. The study was conducted and reported in accordance with the STROBE guidelines.

A non-probabilistic convenience sampling strategy was adopted due to the nationwide scope of the study, the absence of an updated national registry containing veterinarians’ contact information, and the need to reach a large and geographically diverse professional population. Participants were recruited via email invitations using databases provided by the National Association of Small Animal Veterinary Practitioners (Anclivepa Brasil) and Merck Sharp & Dohme Brazil (MSD Brazil).

Eligibility criteria included age ≥18 years (legal adulthood in Brazil) and active engagement in companion-animal practice. Approximately 2,000 professionals accessed and responded to the online questionnaire. Exclusion criteria comprised incomplete questionnaires, duplicate submissions, and respondents not exclusively engaged in small animal practice. After screening for completeness, internal consistency, and compliance with eligibility criteria, 1,472 questionnaires were deemed valid and included in the final analysis.

Restricting the sample to small animal veterinarians aimed to enhance homogeneity of occupational exposure, as companion-animal practice is characterized by frequent client interaction, repeated exposure to euthanasia, and high emotional demands that differ substantially from those in large-animal or mixed practice. This strategy was adopted to strengthen the internal validity of the proposed psychosocial model.

Participation required prior reading of the study objectives and acceptance of the informed consent form. All procedures ensured anonymity and confidentiality of participant data in accordance with ethical standards for research involving human subjects.

Respondents represented diverse specialties within small animal veterinary medicine and were distributed across different geographic regions of Brazil. Data were collected using an online survey administered via SurveyMonkey (SurveyMonkey Inc., San Mateo, CA, USA), which is compatible with desktop and mobile devices. To improve data quality, key variables were set as mandatory fields, logical consistency checks were embedded in the questionnaire, and platform-based restrictions were applied to minimize duplicate submissions. All responses were screened for completeness and internal consistency before analysis. As PLS-SEM does not assume multivariate normality, formal normality testing was not required.

### Instruments

The questionnaire included validated instruments assessing psychological distress, suicidal ideation, coping strategies, compassion fatigue, OS, and WE. Together, these instruments captured individual and organizational psychosocial factors within the SEM framework.

Psychological distress was assessed using the Kessler Psychological Distress Scale (K6) developed by Kessler *et al*. [36]. The scale comprises six items measuring the frequency of symptoms such as nervousness, hopelessness, deep sadness, psychomotor agitation, perceived effort required to perform daily activities, and demotivation. Responses were recorded on a Likert-type scale, with higher scores indicating greater psychological distress. Total scores of 0–7 indicate low distress, 8–12 moderate distress, and ≥13 severe distress. The K6 has demonstrated adequate psychometric properties and has been validated in Brazilian population-based studies [37].

Suicidal ideation was evaluated using four items derived from the Executive Summary of the Merck Animal Health Veterinary Well-being Study [38] and from population-based mental health surveys recommended by the World Health Organization [39]. For each item, respondents selected one of three options: “Yes, in the past 12 months,” “Yes, before the past 12 months,” or “No.” The items addressed key stages of the suicidality continuum, including serious suicidal thoughts, planning, attempts, and help-seeking from the CVV or a healthcare professional. For SEM purposes, responses were dichotomized, with “Yes, in the past 12 months” coded as 1 and all other responses coded as 0. This approach ensured temporal proximity between suicidal ideation and current occupational conditions and is consistent with large-scale mental health research [40, 41].

Coping strategies were assessed using a self-report scale adapted from Folkman and Lazarus [16], focusing on behavioral practices such as physical exercise, socializing with friends, leisure activities, travel, hobbies, and time spent with family. These heterogeneous practices were conceptualized as an emergent construct rather than a reflective latent variable. Responses reflected frequency of engagement on a Likert-type scale.

Compassion fatigue was measured using five behavioral and emotional indicators of empathic exhaustion [42, 43]. Items reflected situations typical of companion-animal practice, including sadness during euthanasia, emotional difficulty associated with patient loss, and feelings of helplessness despite animal suffering. Responses were recorded on a Likert-type scale, with higher scores indicating greater compassion fatigue. Items were adapted from instruments widely applied in healthcare and veterinary contexts with demonstrated psychometric adequacy [42–44].

OS was assessed using four items measuring professional frustration, mismatch between career expectations and actual practice, perceived excessive stress, and limited control over daily activities. These items were developed based on empirical findings from studies on OS among healthcare professionals, including veterinarians [25, 44]. Higher scores reflected greater perceived OS.

The WE was evaluated using two items measuring perceptions of clinical dynamics on a 6-point Likert scale ranging from collaborative/calm to conflicting/chaotic. Items were adapted from organizational climate studies in healthcare settings [38, 45, 46] and captured interpersonal climate, communication quality, and perceived harmony or conflict.

A sociodemographic questionnaire collected information on age, gender, ethnicity, marital status, number of children, geographic region of residence, weekly workload, and monthly income. Income was reported in Brazilian reais and converted to United States dollars using the average exchange rate during the data collection period. All instruments demonstrated adequate internal consistency, with composite reliability (Jöreskog’s ρc) values ranging from 0.757 to 0.827. Measurement instruments and response scale anchors are summarized in Supplementary Table S1.

### Statistical analysis

Statistical power analysis was conducted using G*Power 3.1.9 (Heinrich-Heine-Universität Düsseldorf, Düsseldorf, Germany) [47] to estimate the minimum sample size required to detect significant path coefficients in the proposed model. Parameters included an expected effect size (f²) of 0.02, significance level (α) of 0.05, desired power (1 − β) of 0.95, and six constructs. A minimum sample size of 1,066 participants was required. The final sample (n = 1,472) exceeded this threshold, indicating adequate statistical power.

Missing data were handled using listwise deletion, and only complete cases were included in the analyses. Descriptive statistics were computed using JASP version 0.19.1.0 (University of Amsterdam, Amsterdam, The Netherlands), with significance set at p < 0.05. Categorical variables were summarized using absolute and relative frequencies, while continuous variables were summarized using means, standard deviations, and 95% confidence intervals.

PLS-SEM was performed using ADANCO version 2.4 (Composite Modeling GmbH & Co. KG, Kleve, Germany) to test the proposed theoretical model [48]. Path coefficients were interpreted based on magnitude and statistical significance (*p* < 0.05) [49]. Global model fit was assessed using the standardized root mean square residual (SRMR).

**Figure 1:**
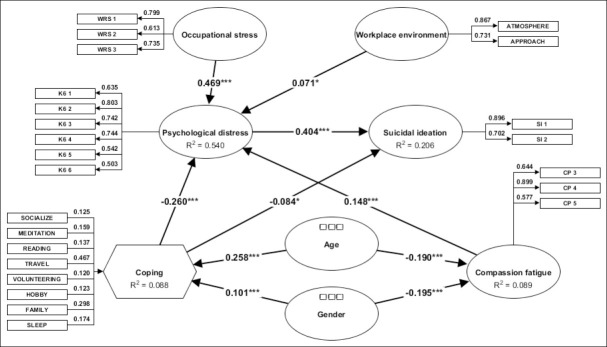
Final parsimonious structural equation model illustrating statistically significant relationships among occupational stress, workplace environment, coping, psychological distress, compassion fatigue, and suicidal ideation (n = 1,472). Path coefficients are shown along the arrows. *p < 0.05, ***p < 0.001.

Seven constructs were included in the structural model: psychological distress, compassion fatigue, suicidal ideation, coping strategies, OS, WE, and workload, with age and gender included as exogenous control variables (Figure 1). Formal multi-group or moderation analyses by age and gender were not conducted because these require prior tests of measurement invariance and balanced subgroup sizes, which were beyond the scope of this study.

Direct and indirect effects were estimated using 5,000 bootstrap resamples with percentile confidence intervals. After establishing adequate measurement properties, the structural model was refined through theory-consistent path trimming, whereby non-significant paths were removed to improve parsimony. Discriminant validity was assessed using the heterotrait–monotrait ratio with a cutoff of 0.85, and internal consistency was evaluated using Jöreskog’s ρc (>0.70). Coefficients of determination (R²) were calculated for endogenous constructs to assess explained variance and overall explanatory quality of the model.

## RESULTS

### Participant characteristics

Most participants were aged 30–39 years (39.8%), followed by those aged 20–29 years (28.3%), indicating a predominantly young sample. Female participation was markedly higher (78.4%), consistent with the feminization of the veterinary profession in Brazil (Table 1), which may have influenced response patterns related to psychological distress in this population. Regarding ethnic and racial composition, most respondents self-identified as white (74.5%), while 23.4% identified as black or brown. More than half of the participants were married (52.2%), followed by single individuals (41.2%), and approximately one-third of the sample (35%) reported having children.

**Table 1:** Work characteristics of the participating veterinarians (n = 1,472).

Characteristic	Category	Frequency	Percentage
Age (years)	20–29	416	28.3%
	30–39	586	39.8%
	40–49	290	19.7%
	50–59	137	9.3%
	≥ 60	43	2.9%
Gender	Female	1154	78.4%
	Male	318	21.6%
Ethnicity	Asian	21	1.4%
	White	1096	74.5%
	Black or brown	344	23.4%
	Others	11	0.7%
Marital status	Single	606	41.2%
	Married	769	52.2%
	Separate	91	6.2%
	Widower	6	0.4%
Children	–	515	35.0%
Macroregion	Center-West	84	5.7%
	North	42	2.9%
	Northeast	196	13.3%
	South	272	18.5%
	Southeast	878	59.6%
Monthly income (USD)^a^	Up to 640	641	43.5%
	641–1165	466	31.7%
	1166–2330	251	17.1%
	≥ 2331	114	7.7%

a Brazilian minimum wage in 2022: USD 220

### Geographic distribution and income profile

The geographic distribution showed a high concentration of professionals in the Southeast (59.6%) and South (18.5%) regions, reflecting the greater density of veterinary services and establishments in these areas. In terms of monthly income, 43.5% of participants reported earnings of up to US$ 640 (US$ 7,680 annually), corresponding to the national minimum wage in 2022, suggesting that a substantial proportion of the sample was economically vulnerable.

### Suicidal ideation and related behaviors

When considering experiences occurring before the past 12 months, a subset of participants reported lifetime suicidality that could not be directly attributed to their current occupational context due to uncertainty regarding timing. Specifically, 18.8% reported serious suicidal thoughts, 11.1% reported suicide planning, and 5.4% reported suicide attempts. Additionally, 4.9% reported having sought support from the CVV or a mental health professional during earlier periods.

Within the past 12 months, 19.2% of participants reported suicidal ideation, 9.0% reported suicide planning, 2.4% reported suicide attempts, and 6.7% reported seeking professional or hotline support (Table 2).

**Table 2:** Response frequency to questions related to suicidal ideation among veterinarians (n = 1,472).

Item	Frequency	Percentage
1. Have seriously thought about trying to kill oneself at some point.	282	19.2%
2. Have made plans to kill oneself.	133	9.0%
3. Have attempted to kill oneself.	36	2.4%
4. Contacted the CVV* or any mental healthcare professional.	98	6.7%

* CVV = Center for Life Valorization.

### Coping strategies, psychological distress, and compassion fatigue

As shown in Table 3, the most frequently adopted coping strategies were spending time with family (M = 3.18; SD = 0.79), sleeping at least eight hours per night (M = 2.75; SD = 0.99), and socializing with friends (M = 2.72; SD = 0.82). Psychological distress assessed using the K6 scale revealed the highest mean scores for the items “feeling that everything was an effort” (M = 2.25) and “feeling restless” (M = 2.22), indicating persistent emotional symptoms. Compassion fatigue scores were particularly elevated for sadness associated with euthanasia procedures (M = 3.97) and difficulty coping with patient loss (M = 3.42), reflecting substantial emotional exhaustion.

**Table 3:** Means and standard deviations of psychological distress (K6), coping strategies, and compassion fatigue scores (n = 1,472).

Variable	Item	Score range	M	SD	95% CI
Coping strategies	1. Perform physical exercise	1–4	2.52	1.155	2.47–2.58
	2. Yoga class		1.19	0.584	1.16–1.22
	3. Socialize with friends		2.72	0.822	2.67–2.76
	4. Meditate		1.51	0.861	1.46–1.55
	5. Reading for pleasure		2.24	1.003	2.19–2.29
	6. Travel for pleasure		2.23	0.840	2.18–2.27
	7. Volunteering		1.68	0.909	1.63–1.73
	8. Practice any hobby		2.43	1.002	2.38–2.48
	9. Spending time with family		3.18	0.788	3.14–3.22
	10. Sleep for at least 8 hours per night		2.75	0.991	2.70–2.80
Psychological distress (K6)	1. Nervous	0–4	1.94	1.084	1.89–2.00
	2. Hopeless		1.59	1.261	1.52–1.65
	3. So sad, nothing could cheer you up		1.18	1.139	1.12–1.23
	4. Worthless		1.26	1.232	1.20–1.33
	5. Restless or antsy		2.22	1.156	2.16–2.28
	6. That everything was an effort		2.25	1.070	2.20–2.31
Compassion fatigue	1. Euthanasia causes me sadness	1–5	3.97	1.033	3.92–4.02
	2. Finding the right moment to suggest euthanasia to the patient is difficult		3.28	1.161	3.22–3.34
	3. I find it difficult to cope with losing patients		3.42	1.111	3.37–3.48
	4. I feel helpless when I lose a patient		3.32	1.171	3.26–3.38
	5. I find it difficult to communicate bad news		3.23	1.169	3.17–3.29

M = Mean, SD = Standard deviation, CI = Confidence interval

### WE and OS

Perceptions of the WE were moderate, with a mean score of 3.00 for clinic atmosphere (ranging from calm to chaotic) and 2.68 for work approach (ranging from collaborative to conflictive). Regarding OS, the item “the work is extremely stressful” showed the highest mean score (M = 2.94), highlighting a relevant emotional burden associated with professional practice (Table 4).

**Table 4:** Mean ± standard deviation values of occupational stress, workload, and workplace environment scores among participating veterinarians (n = 1,472).

Variable	Item	Score range	M	SD	95% CI
Occupational stress	1. Work is frustrating	1–4	2.58	0.945	2.53–2.63
	2. The practice has not met my expectations		2.44	0.973	2.39–2.49
	3. The job is extremely stressful		2.94	0.910	2.89–2.99
	4. There are few stressful things at work		1.91	0.919	1.86–1.96
Workload	Hours worked per week	–	42.9	15.18	42.1–43.6
Workplace environment	1. Clinic atmosphere (1 = calm; 6 = chaotic)	1–6	3.00	1.264	2.93–3.06
	2. Approach to work in your clinic (1 = collaborative; 6 = conflicting)		2.68	1.304	2.61–2.75

M = Mean, SD = Standard deviation, CI = Confidence interval

### Measurement model assessment

Supplementary Figure S1 presents the initial theoretical model derived from the literature, whereas Figure 1 shows the final adjusted model, in which paths not supported by the data (p > 0.05) were removed to improve parsimony and interpretability. Discriminant validity was supported by heterotrait–monotrait ratio values below the recommended cutoff of 0.85 for all constructs (Table 5). Convergent validity, assessed using average variance extracted, showed most constructs with values close to or exceeding 0.50. Internal consistency was satisfactory, with composite reliability (Jöreskog’s ρc) ranging from 0.757 to 0.827 and Cronbach’s alpha values between 0.749 and 0.823, indicating adequate reliability and robustness of the measurement model. The final PLS-SEM model demonstrated acceptable global fit, with an SRMR value of 0.073. Multicollinearity diagnostics indicated no concerns, as all variance inflation factor values were below 3.3.

**Table 5:** Measurement model evaluation showing discriminant validity using HTMT, convergent validity through, and internal consistency reliability of reflective constructs (n = 1,472).

Construct	HTMT (1)	HTMT (2)	HTMT (3)	HTMT (4)	Jöreskog’s ρc	AVE	Cronbach’s alpha
1. Suicidal ideation	–	–	–	–	0.784	0.648	0.773
2. Psychological distress	0.447	–	–	–	0.827	0.450	0.823
3. Compassion fatigue	0.151	0.392	–	–	0.757	0.519	0.749
4. Workplace environment	0.196	0.385	0.196	–	0.781	0.643	0.776
5. Occupational stress	0.305	0.678	0.364	0.455	0.761	0.518	0.757

HTMT = Heterotrait–monotrait ratio of correlations, AVE = Average variance extracted.

### Structural model results

Psychological distress exerted a strong positive effect on suicidal ideation (β = 0.40; p < 0.001), representing the most robust predictor in the model (Table 6). OS showed a significant positive association with psychological distress (β = 0.47; p < 0.001), indicating a substantial impact of work-related pressure on mental health. Compassion fatigue was also positively associated with psychological distress (β = 0.15; p < 0.001), suggesting that prolonged emotional involvement with patient suffering contributed to worsening distress. The WE had a smaller but significant effect on psychological distress (β = 0.07; p = 0.028), indicating a moderate influence of contextual practice factors.

**Table 6:** Structural equation model results showing direct and indirect effects among study constructs based on bootstrap analysis (n = 1,472).

Effect	Original coefficient	M	SE	t	P (2-sided)	2.5%	97.5%
Direct effects inference							
Psychological distress → Suicidal ideation	0.40	0.40	0.04	10.60	< 0.001	0.33	0.48
Compassion fatigue → Psychological distress	0.15	0.15	0.03	5.23	< 0.001	0.09	0.20
Coping → Suicidal ideation	−0.08	−0.09	0.03	−2.48	0.013	−0.15	−0.02
Coping → Psychological distress	−0.26	−0.26	0.03	−9.11	< 0.001	−0.32	−0.21
Workplace environment → Psychological distress	0.07	0.07	0.03	2.20	0.028	0.01	0.13
Occupational stress → Psychological distress	0.47	0.47	0.03	12.41	< 0.001	0.40	0.54
Age → Compassion fatigue	−0.19	−0.19	0.03	−6.29	< 0.001	−0.25	−0.13
Age → Coping	0.26	0.26	0.04	7.50	< 0.001	0.19	0.32
Gender → Compassion fatigue	−0.19	−0.19	0.03	−6.47	< 0.001	−0.25	−0.14
Gender → Coping	0.10	0.10	0.03	3.43	< 0.001	0.04	0.16
Indirect effects inference							
Compassion fatigue → Suicidal ideation	0.06	0.06	0.01	4.73	< 0.001	0.04	0.09
Coping → Suicidal ideation	−0.10	−0.11	0.02	−6.88	< 0.001	−0.14	−0.08
Workplace environment → Suicidal ideation	0.03	0.03	0.01	2.16	0.030	0.00	0.06
Occupational stress → Suicidal ideation	0.19	0.19	0.02	7.87	< 0.001	0.14	0.24
Age → Suicidal ideation	−0.06	−0.06	0.01	−5.91	< 0.001	−0.08	−0.08
Age → Psychological distress	−0.10	−0.09	0.01	−7.17	< 0.001	−0.12	−0.04
Gender → Suicidal ideation	−0.03	−0.03	0.01	−4.14	< 0.001	−0.05	−0.02
Gender → Psychological distress	−0.06	−0.06	0.01	−4.72	< 0.001	−0.08	−0.03

M = Mean, SE = Standard error, t = t-value, p = Probability value, 2.5% and 97.5% = Lower and upper bounds of the percentile bootstrap 95% confidence interval.

Coping strategies demonstrated a protective effect on psychological distress (β = −0.26; p < 0.001) and a direct protective effect on suicidal ideation (β = −0.08; p = 0.013), indicating that adaptive coping reduced emotional burden and mitigated suicidal thoughts. Workload showed no significant association with psychological distress or suicidal ideation, suggesting that qualitative aspects of the work environment may be more influential than the number of hours worked.

### Sociodemographic effects and mediation

Age was negatively associated with compassion fatigue (β = −0.19; p < 0.001) and psychological distress (β = −0.09; p < 0.001) and showed a negative indirect effect on suicidal ideation (β = −0.06; p < 0.001), indicating higher vulnerability among younger professionals. Female gender was associated with higher compassion fatigue (β = −0.19; p < 0.001) and lower engagement in adaptive coping strategies (β = 0.10; p = 0.001). These associations were linked to higher psychological distress (β = −0.06; p < 0.001) and, indirectly, to suicidal ideation (β = −0.03; p < 0.001).

Among indirect effects, coping strategies showed a substantial mediated protective effect on suicidal ideation through reduced psychological distress (indirect β = −0.11; p < 0.001). OS (β = 0.19; p < 0.001) and WE (β = 0.03; p = 0.030) also indirectly influenced suicidal ideation via increased psychological distress, supporting robust mediation pathways.

### Explained variance and effect sizes

The coefficients of determination for the endogenous constructs were 0.206 for suicidal ideation, 0.540 for psychological distress, 0.089 for compassion fatigue, and 0.088 for coping strategies. Effect size analysis indicated that OS had the largest contribution to psychological distress (f² = 0.297), followed by the effect of psychological distress on suicidal ideation (f² = 0.148) (Table 7). Coping strategies showed a moderate contribution to psychological distress (f² = 0.113), whereas the remaining paths demonstrated small effect sizes.

**Table 7:** Effect size (Cohen’s f²) values for the direct relationships in the structural equation model.

Direct path	f²
Occupational stress → Psychological distress	0.297
Psychological distress → Suicidal ideation	0.148
Coping → Psychological distress	0.113
Compassion fatigue → Psychological distress	0.041
Workplace environment → Psychological distress	0.009
Coping → Suicidal ideation	0.006
Age → Coping	0.070
Gender → Coping	0.011

f² = Cohen’s effect size for structural paths, where 0.02 = Small effect, 0.15 = Medium effect, 0.35 = Large effect.

## DISCUSSION

### Overview of principal findings

This study represents the first large-scale SEM-based investigation of suicidal ideation among veterinarians in Brazil, integrating coping strategies, CF, WE, and OS into a single explanatory framework. Grounded in the JD-R model and ITS, the findings demonstrate that suicidal ideation among Brazilian veterinarians is a multifactorial phenomenon shaped by emotional, contextual, and individual determinants. This interpretation is consistent with suicidology frameworks that emphasize the multidimensional nature of suicidal behavior and the need for interdisciplinary prevention approaches [50, 51]. In line with broader evidence, women were more likely to report emotional symptoms, help-seeking behaviors, and participation in mental health surveys, patterns commonly attributed to sociocultural norms and gender differences in emotional expression and coping [52–55]. Although age and gender showed statistically significant direct and indirect associations with several constructs, these effects represent adjusted main effects within the overall structural model and should not be interpreted as moderation or subgroup-specific mechanisms, which would require formal interaction analyses.

### Mediating role of psychological distress

PLS-SEM analyses highlighted the central mediating role of psychological distress in the associations between CF, OS, WE, coping strategies, and suicidal ideation. These findings corroborate international literature identifying chronic psychological distress as a key proximal factor underlying healthcare professionals’ vulnerability to suicidal behavior [40, 51, 56]. Within the ITS, psychological distress appears to function as an emotional mechanism through which distal occupational stressors are translated into suicidal ideation, rather than suicidal thoughts emerging directly from contextual exposures.

### International and national context

The present results are consistent with international evidence reporting elevated psychological distress and suicide-related outcomes among veterinarians, particularly those working in small animal practice in the United States, the United Kingdom, Australia, and Europe [2, 5, 6]. Similar psychosocial determinants, including OS, CF, and organizational factors, have been identified across these settings [3, 5, 7]. The mediating role of psychological distress observed in this study aligns with international suicide-prevention frameworks that conceptualize distress as a proximal pathway linking occupational stressors to suicidal ideation [50, 51].

At the same time, the Brazilian context presents specific cultural and socioeconomic characteristics that may intensify vulnerability, including the feminization of the veterinary workforce, comparatively low income levels, high emotional demands associated with companion-animal care and euthanasia, and limited institutional mental health support. These factors may partly explain the high prevalence of suicidal ideation observed and underscore the need to integrate international prevention models with locally tailored strategies sensitive to Brazilian veterinary practice realities [7, 9].

### Suicidal ideation within the suicidality continuum

The distinction between suicidal ideation and suicidal behavior lies in the presence of action. Whereas behavior involves execution, attempt, or active preparation, ideation remains cognitive in nature. The presence of ideation accompanied by planning, reported by 9.0% of participants in the past 12 months, represents a markedly elevated risk of progression toward suicidal behavior and warrants immediate clinical attention [50]. This distinction is central to the ITS, which conceptualizes suicidal ideation as an early yet clinically critical stage of the suicidality continuum, particularly when coupled with elevated psychological distress.

### OS and WE

Within the JD-R framework, the strong association between OS and psychological distress (β = 0.47) reinforces the premise that work environments characterized by high emotional demands, limited autonomy, and inadequate recognition exacerbate distress. Veterinarians experience pressures comparable to those reported in other healthcare professions, although direct comparative studies remain limited. In veterinary practice, low social and economic recognition has been identified as a relevant contributor to frustration and hopelessness [2, 25, 27]. By integrating these occupational determinants into a unified model, this study advances understanding of the mechanisms underlying mental illness in veterinary medicine. The model demonstrated moderate explanatory power for suicidal ideation and psychological distress and smaller but meaningful effects for other mediating variables.

### Compassion fatigue as an indirect pathway

CF showed a significant positive association with psychological distress (β = 0.15), highlighting the impact of prolonged emotional engagement with animal suffering and owner distress, particularly in euthanasia-related contexts. This finding is consistent with prior evidence linking empathic fatigue to burnout, absenteeism, and suicidal ideation among healthcare professionals [20, 22, 23]. However, the absence of a direct effect of CF on suicidal ideation in the final model contrasts with some earlier studies, especially those focusing on euthanasia-related stress [50, 57].

A plausible explanation is that CF influences suicidal ideation indirectly through psychological distress. Continuous exposure to suffering may contribute to cumulative emotional burden, which then acts as a proximal driver of suicidal ideation. This mediation pathway may have attenuated the direct effect of CF without diminishing its structural relevance, aligning with contemporary health psychology and suicidology models emphasizing indirect and emotionally mediated trajectories of suicide risk [51, 58].

### Protective role of coping strategies

Adaptive coping strategies demonstrated both direct (β = −0.08) and indirect (β = −0.11) protective effects against suicidal ideation through reductions in psychological distress. Within the JD-R framework, these strategies function as personal resources that buffer the impact of occupational demands. Activities such as social interaction, leisure, adequate rest, and physical exercise are well established as protective mechanisms in high-demand professional contexts [19, 29, 59]. Nonetheless, the low R² value for coping strategies suggests that although these behaviors are protective at the individual level, their adoption may be constrained by organizational and structural barriers, including excessive workload, insufficient breaks, and occupational overload.

### Workload and qualitative job demands

Weekly workload was not significantly associated with psychological distress or suicidal ideation. Although long working hours have been linked to burnout and suicide risk in other healthcare contexts [2, 60, 61], the present findings suggest that qualitative aspects of WE, such as organizational culture, emotional support, and professional autonomy, may exert a stronger influence on mental health outcomes in Brazilian companion-animal practice.

### Sociodemographic influences

Younger age was associated with higher CF and psychological distress, consistent with evidence indicating greater emotional vulnerability earlier in professional careers [2, 62, 63]. Female gender was also associated with higher distress and CF, in line with literature documenting greater empathic demands and distinct help-seeking patterns among women in healthcare professions [52, 64, 65]. These disparities highlight the need for equitable, targeted interventions and reinforce the relevance of psychosocial workplace factors in shaping mental health outcomes.

Importantly, age and gender did not show direct associations with suicidal ideation, with their effects occurring indirectly via psychological distress. This finding partially diverges from international literature reporting higher suicidal ideation and suicide mortality among men [5, 66]. In the present model, emotional variables may have absorbed these effects, explaining the absence of direct associations.

### Implications for prevention and policy

By integrating the JD-R model and ITS, these findings indicate that effective prevention strategies should simultaneously address occupational demands, strengthen personal and organizational resources, and reduce psychological distress as a proximal driver of suicidal ideation. At the clinical level, interventions enhancing social support, professional autonomy, and emotional assistance may mitigate distress. At the institutional level, professional councils and associations may play a key role in promoting mental health training, adaptive coping, and awareness initiatives. Routine screening for psychological distress may facilitate early identification of clinicians at elevated risk.

At the public policy level, these results support the inclusion of veterinarians in occupational mental health surveillance and suicide-prevention frameworks within the Brazilian Ministry of Health. Moreover, the study aligns with the United Nations Sustainable Development Goal 3 (Good Health and Well-being), reinforcing the prioritization of mental health promotion among healthcare-related professionals as a public health imperative.

### Methodological limitations

Despite the robustness of the findings, several methodological limitations should be acknowledged. First, the cross-sectional design precludes causal inference among the analyzed constructs, allowing only correlational interpretations. Second, the non-probabilistic sampling strategy and voluntary participation may have introduced selection and non-response bias, as only a proportion of the approximately 20,000 invited professionals completed the survey. Because all data were collected using online self-report instruments, the findings may also be influenced by self-report and social desirability biases.

Recruitment through industry-linked databases (MSD Brazil) and the exclusive use of an online data collection format may have further favored participation by individuals who were more sensitized, available, or interested in mental health–related topics. In addition, the sample was predominantly composed of women and professionals from the Southeast region of Brazil, which may limit national representativeness and restrict the generalizability of the findings to veterinarians working in other regions or practice settings.

### Analytical and design considerations

These limitations do not diminish the contribution of the present study to advancing understanding of psychosocial determinants of mental health among Brazilian veterinarians. By integrating multiple occupational, emotional, and individual variables into a single SEM framework, the study provides robust empirical evidence for contextually sensitive preventive strategies by elucidating direct and indirect pathways linking WE, coping strategies, psychological distress, and suicidal ideation.

Although age and gender were included as covariates in the structural model, formal multigroup or interaction analyses were not conducted. Such analyses require prior tests of measurement invariance and more balanced subgroup distributions, which were beyond the scope of the present investigation. Moreover, the study was primarily explanatory and theory-driven rather than prediction-oriented. Consequently, predictive validation procedures, including PLSpredict, cross-validation, or comparisons with alternative structural models, were not performed. While these approaches may enhance assessment of out-of-sample predictive performance, they were considered outside the scope of the current study.

### Directions for future research

Future studies should adopt longitudinal designs to clarify causal relationships among occupational stressors, emotional processes, and suicidal ideation. Further research is also warranted to examine regional and cultural variations in psychosocial risk factors and to assess the effectiveness of targeted interventions, such as structured emotional support programs, coping strategy training, and organizational policy modifications, in reducing psychological distress and suicidal ideation within veterinary clinical practice.

## CONCLUSION

This study provides robust evidence that psychological distress is a central mechanism linking occupational and psychosocial factors to suicidal ideation among veterinarians working in companion-animal practice in Brazil. Using an integrated SEM grounded in the JD-R model and ITS, the findings demonstrate that OS, CF, and WE significantly increase psychological distress, whereas adaptive coping strategies exert both direct and indirect protective effects. Psychological distress emerged as the strongest predictor of suicidal ideation, explaining a substantial proportion of its variance, while indirect effects confirmed its mediating role between occupational stressors and suicidal outcomes. These results underscore that suicidal ideation in veterinarians is not driven by isolated factors but arises from the convergence of emotional burden, organizational context, and individual coping capacity.

The findings have important implications for veterinary practice, institutional policy, and public health. At the clinical level, interventions aimed at reducing OS and improving WE—such as fostering supportive team dynamics, enhancing professional autonomy, and improving communication—may substantially mitigate psychological distress. The protective role of coping strategies highlights the need to facilitate access to rest, social support, and leisure opportunities within clinical routines. At the institutional level, professional councils and associations can contribute by implementing mental health training, promoting awareness of CF, and integrating routine screening for psychological distress as part of occupational health programs. At the policy level, the results support the inclusion of veterinarians within national occupational mental health and suicide-prevention frameworks, enabling earlier identification of high-risk professionals and the development of targeted, context-sensitive interventions.

Key strengths of this study include its large nationwide sample, focus on veterinarians exclusively engaged in companion-animal practice, and use of a theory-driven SEM approach that simultaneously examined multiple direct and indirect pathways. The integration of JD-R and ITS allowed for a comprehensive assessment of how occupational demands, emotional processes, and personal resources interact to influence suicidal ideation. The identification of psychological distress as a central mediating construct represents a meaningful advance in understanding suicide risk mechanisms in veterinary medicine.

In conclusion, this study highlights psychological distress as a critical and actionable target for suicide prevention in veterinary medicine. Addressing OS, CF, and WE while strengthening adaptive coping strategies may substantially reduce suicidal ideation among veterinarians. These findings reinforce the need for coordinated, multi-level prevention efforts involving clinics, professional organizations, and policymakers. Prioritizing veterinarians’ mental health is essential not only for individual well-being but also for sustaining a resilient and ethical veterinary workforce.

## DATA AVAILABILITY

The supplementary data can be made available from the corresponding author upon request.

## AUTHORS´ CONTRIBUTIONS

BG: Conceptualization, investigation, supervision, and drafting of the manuscript. JLP: Conceptualization, investigation, and drafting of the manuscript. AS: Conceptualized and drafted the manuscript. KS: Investigation and drafting of the manuscript. HLC and AR: Investigation and drafting of the manuscript. SU: Conceptualization and drafting of the manuscript. All authors have read and approved the final version of the manuscript.

## COMPETING INTERESTS

The authors declare that they have no competing interests.

## PUBLISHER’S NOTE

Veterinary World remains neutral with regard to jurisdictional claims in the published institutional affiliations.
